# *BAIAP2* exhibits association to childhood ADHD especially predominantly inattentive subtype in Chinese Han subjects

**DOI:** 10.1186/1744-9081-9-48

**Published:** 2013-12-30

**Authors:** Lu Liu, Li Sun, Ze-Hua Li, Hai-Mei Li, Li-Ping Wei, Yu-Feng Wang, Qiu-Jin Qian

**Affiliations:** 1 Peking University Sixth Hospital/Institute of Mental Health, Beijing 100191, China; 2 Key Laboratory of Mental Health, Ministry of Health, Peking University, Beijing 100191, China; 3 Center for Bioinformatics, National Laboratory of Protein Engineering and Plant Genetic Engineering, College of Life Sciences, Peking University, Beijing 100871, China

**Keywords:** Attention-deficit/hyperactivity disorder (ADHD), *BAIAP2*, Hemispheric asymmetry, Subtype

## Abstract

**Background:**

Attention-deficit/hyperactivity disorder (ADHD) is a common chronic neurodevelopmental disorder with a high heritability. Much evidence of hemisphere asymmetry has been found for ADHD probands from behavioral level, electrophysiological level and brain morphology. One previous research has reported possible association between *BAIAP2*, which is asymmetrically expressed in the two cerebral hemispheres, with ADHD in European population. The present study aimed to investigate the association between *BAIAP2* and ADHD in Chinese Han subjects.

**Methods:**

A total of 1,397 ADHD trios comprised of one ADHD proband and their parents were included for family-based association tests. Independent 569 ADHD cases and 957 normal controls were included for case-control studies. Diagnosis was performed according to the DSM-IV criteria. Nine single nucleotide polymorphisms (SNPs) of *BAIAP2* were chosen and performed genotyping for both family-based and case-control association studies.

**Results:**

Transmission disequilibrium tests (TDTs) for family-based association studies showed significant association between the CA haplotype comprised by rs3934492 and rs9901648 with predominantly inattentive type (ADHD-I). For case-control study, chi-square tests provided evidence for the contribution of SNP rs4969239, rs3934492 and rs4969385 to ADHD and its two clinical subtypes, ADHD-I and ADHD-C. However, only the associations for ADHD and ADHD-I retained significant after corrections for multiplicity or logistic regression analyses adjusting the potential confounding effect of gender and age.

**Conclusions:**

These above results indicated the possible involvement of *BAIAP2* in the etiology of ADHD, especially ADHD-I.

## Background

Attention-deficit/hyperactivity disorder (ADHD) is a common neurodevelopmental disorder. The high heritability of approximately 0.76 [1] has suggested the important role of genetic factors in its etiology.

The typical development of human cerebral asymmetry has profound effect on the normal lateralization of cognitive and motor functions, such as language and handedness. Several studies have suggested the possible link of the disturbance of cerebral asymmetry with the pathogenesis of neurodevelopmental disorders [2], such as schizophrenia [3], autism [4] and ADHD [5].

The non-right-handedness, especially mixed-handedness, might be a risk indicator for the symptom severity of inattention in ADHD [6]. One of our previous behavioral studies indicated that ADHD children showed an atypical pattern of right hemisphere in conflict control task compared to controls [7]. Another study demonstrated that the direction of spatially asymmetrical interference effects in ADHD was opposite to controls, indicating disruption within right hemisphere attentional networks [8]. The structural imaging findings in ADHD has demonstrated volumetric reductions in total and right cerebral, right caudate, cerebellar and corpus callosum [9]. ADHD adults had thinner cortex in the cortical networks, especially in the right hemisphere involving inferior parietal lobule, dorsolateral prefrontal and anterior cingulate cortices [10]. In addition, some evidence has showed that the critical feature of ADHD is the delayed maturation not only of prefrontal cortical thickness but also of cortical surface area, especially the right hemispheric lobes [11,12]. The degree of rightward volumetric asymmetry in caudate nucleus might predict the severity of inattentive symptoms of ADHD [13]. An fMRI study of ADHD children showed under-activation of the right caudate nucleus and inferior parieta cortex [14].

The gene *BAIAP2*, which is located on 17q25 and encodes brain-specific angiogenesis inhibitor 1-associated protein 2 (BAIAP2), has been suggested to be involved in cerebral asymmetry [15]. Recently, a genetic study of adulthood ADHD in two independent European populations suggested an association of *BAIAP2* with ADHD, supplying genetic evidence of abnormal left-right brain asymmetries with this disorder [16]. In addition, a genome-wide association study (GWAS) has indicated nominal association between *BAIAP2* and its isoform *BAIAP2L1* with ADHD [17]. Moreover, methamphetamine, one of psychostimulants being considered as first-line pharmacological treatments for ADHD patients, has enhanced the expression of *BAIAP2* in rat cerebral cortices [18]. *BAIAP2* has also been found to confer risk for autism spectrum disorders (ASD), which shared some genetic risk factors with ADHD [19].

For the potentially important role of cerebral asymmetry in the pathophysiology of ADHD, *BAIAP2* has been suggested to be one of novel candidate genes for ADHD, but need more work for replication [20]. Our present study is to investigate the relationship between *BAIAP2* and ADHD in Chinese Han subjects.

## Methods

### Subjects

All ADHD cases were recruited from child psychiatric clinics of Peking University Institute of Mental Health. Diagnosis was performed according to the DSM-IV criteria by experienced child psychiatrists, using the Clinical Diagnostic Interview Scale (CDIS) [21], which was translated into Chinese by our groups before [22,23]. The CDIS assesses the three DSM-IV subtypes of ADHD, including ADHD inattentive type (ADHD-I), ADHD hyperactive-impulsive type (ADHD-HI) and ADHD combined type (ADHD-C). It was also used to evaluate comorbidities, including oppositional defiant disorder (ODD), tic disorder (TD), learning disorder (LD), etc. The diagnosis of LD is just based on a brief parents’ report of the general academic achievement, but not including detailed assessment for reading, writing or arithmetic abilities. So dyslexia, dysgraphia or dyscalculia was not defined in our current study. All cases were of Chinese Han descent, with age between 6 and 16 years, and full-scale estimated IQ > 70. Any major neurological disorders, a diagnosis of schizophrenia, pervasive development disorder, epilepsy, mental retardation or other brain disorders were excluded. Finally, a total of 1,966 ADHD probands were included. Among these cases, 1,397 ADHD probands, along with their parents, constituted trios for family-based association analyses. The other independent 569 ADHD cases were included for case-control study.

The control sample consisted of 957 subjects from local elementary schools, healthy blood donors from the blood center of the First Hospital of Peking University, and healthy volunteers at our institute. ADHD, other major psychiatric disorders, family history of psychosis, severe physical diseases and substance abuse were excluded (more details have been described in our previously published article [24]). Demographic and clinical characteristics of both ADHD cases and control sample are shown in Table 1.

**Table 1:** Demographic and clinical characteristics of sample

	**Family-based study**	**Case-control study**		
	**ADHD (trios)**	**ADHD (indep.)**	**Controls**	***P *** **values**
**N**	1397	569	957	
**Mean Age (SD)**	9.9 (2.5)	10.5 (2.6)	15.4 (8.5)	< 0.001
**N Male (%)**	1171 (83.9)	465 (81.7)	617 (64.5)	< 0.001
**N subtypes (%)**				
**Inattentive**	706 (50.5)	295 (51.8)		
**Hyperactive**-**impulsive**	71 (5.1)	22 (3.9)		
**Combined**	620 (44.4)	252 (44.3)		

*Abbreviations:ADHD* attention-deficit/hyperactivity disorder, *SD* standard deviation, *ADHD* (indep.) independent ADHD cases for case-control study, not including ADHD probands from trios sample.

All subjects were treated according to the Declaration of Helsinki and this work was approved by the Ethics Committee of Peking University Health Science Center. Written informed consent was obtained from each subjects or parents of children.

### DNA isolation

Peripheral blood was collected for all subjects included in this study. Then genomic DNA was extracted following the standard protocols using E.Z.N.A.™ Blood DNA Kits (Omega Bio-tek Inc., Doraville, GA).

### SNP selection and genotyping

For selection of single nucleotide polymorphisms (SNPs), Haploview version 4.2 was used to pick up tag SNPs based on the CHB database from Hapmap (http://www.hapmap.org). Threshold limit was set of *r*^*2*^ > 0.80 for all SNPs with minor allele frequency (MAF) > 0.25. Thirteen tag SNPs were chosen with these criteria. However, only eight tag SNPs were included for genotyping and further association analyses. The other five SNPs were not selected for the location at the same haplotype block or strong to moderate linkage disequilibrium (LD) with those selected SNPs (Table 2). We also included an additional SNP rs4969385 located on intron 6, which was found to be potentially associated with adult ADHD in Spanish population [16].

**Table 2:** Selection of SNPs

**No.**	**SNP marker**	**Source**^**a**^	**Included**^**b**^	**Location**	**Allele**
SNP 1	rs4969239	Tag SNP	Y	Intron 1	A/G
SNP 2	rs4969358	Tag SNP	Y	Intron 1	A/C
SNP 3	rs6565531	Tag SNP	Y	Intron 1	A/G
SNP 4	rs8079626	Tag SNP	Y	Intron 1	A/G
SNP 5	rs3934492	Tag SNP	Y	Intron 3	C/G
SNP 6	rs9901648	Tag SNP	Y	Intron 3	A/G
SNP 7	rs4076037	Tag SNP	Y	Intron 3	A/G
SNP 8	rs8066330	Tag SNP	Y	Intron 3	C/T
SNP 9	rs4969385	reported	Y	Intron 6	C/T
	rs8067235	Tag SNP	N, located on the same block with rs6565531(*D*’ = 1, *r*^*2*^ = 0.642)		
	rs6565532	Tag SNP	N, located on the same block with rs6565531 (*D*’ = 1, *r*^*2*^ = 0.798)		
	rs8070741	Tag SNP	N, with moderate LD with rs9901648 (*D*’ = 0.896, *r*^*2*^ = 0.803)		
	rs11657997	Tag SNP	N, with moderate LD with rs9901648 (*D*’ = 1, *r*^*2*^ = 0.783)		
	rs4969355	Tag SNP	N, located on the same block with rs4969239 (*D*’ = 1, *r*^*2*^ = 0.764) and rs4969359 (*D*’ = 1,*r*^*2*^ = 0.783)		

*Abbreviation:LD* linkage disequilibrium.

^a^tag SNP, pick up using Haploview based on the *CHB* database from Hapmap, with an *r*^*2*^ threshold of 0.80 from all SNPs with minor alleles frequency (MAF) > 0.25; reported, which has been studied and shown potential association with adult ADHD in Spanish population [16].

^b^Y, included for further genotyping and analyses; N, not included for further genotyping.

Genotyping for all SNPs were carried out on an ABI 7900-HT instrument (Applied Biosystems, Foster City, USA), using Taqman allelic genotyping assays [25] and following the standard protocol as described by our previous report [26]. The SDS version 2.3 software was used for genotype identification. For quality control, firstly, two to four non-template controls (NTC) were set on each 384-well plate with no genotype called. Secondly, 3% samples were selected randomly and genotyped for the same SNP by different experimenters, indicating the concordance rate of 100%. Call rates for all SNPs were ranging from 98.8% to 99.5%.

### Statistical analyses

**Figure 1:**
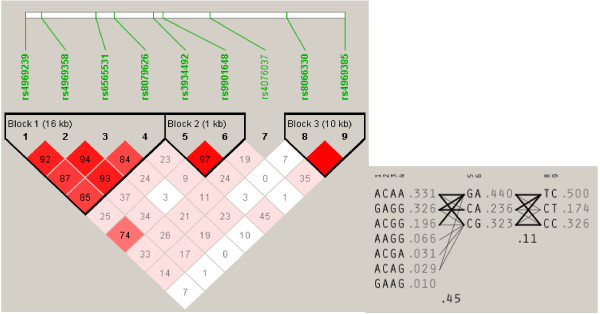
**Linkage disequilibrium (LD) plot of *BAIAP2 *estimated by Haploview software.** It displayed LD value in *D’* (i.e., 99 indicated *D’* of 0.99. Squares with no number indicates a *D’* of 1). Generated haplotypes were listed on the right for each of three LD blocks with frequencies.

Calculation of MAF and analysis of Hardy-Weinberg equilibrium (HWE) were performed using Haploview 4.2 software for cases and controls separately, showing no departure from HWE for any SNP (*P*-value from 0.178 to 0.921). The MAFs for all SNPs were from 0.162 to 0.499. Haploview software was also used to estimate the linkage disequilibrium (LD) of all SNPs and generate haplotype blocks which will be used for multi-marker haplotype-based association tests [27]. As shown by Figure 1, there were three haplotype blocks generated including Block 1 comprised of SNP 1–4, Block 2 comprised of SNP 5–6 and Block 3 comprised of SNP 8–9. However, the SNP7 rs4076037 was not included into any block and subsequently not included for multi-marker analyses. For Block 1, only three of seven generated haplotypes (ACAA, GAGG and ACGG) were used for haplotype analyses, which captured 85.3% of the genetic variance in the investigated four SNPs, while the other four haplotypes with frequencies < 0.1 were not included.

For family-based association study, TDT tests were conducted using Haploview to investigate whether there was biased-transmission of alleles or haplotypes in ADHD trios. For case-control study, chi-square tests were conducted using Haploview for both single-marker and multi-marker haplotype-based analyses, to compare the frequencies of alleles and haplotypes between ADHD cases and controls. In addition, comparison of the genotype frequencies was also performed for case-control statistics using SPSS software. Once there was nominally significant association appeared either for alleles or genotypes under additive model (nominal *P* < 0.05), further analyses for genotypes based on dominant and recessive models were conducted [16]. As indicated in Table 1, the comparisons of distribution of gender and age between two groups showed statistical significance. Then, we further developed logistic regression analyses to control the potential confounding effect of gender and age.

For multiple testing corrections of single-marker analyses, Bonferroni corrections were conducted. We firstly used SNPSpD software [28] to consider the inter-correlation of SNPs for multiple testing of SNPs in LD with each other. For our current study, the effective number of independent marker loci yielded by SNPSpD was 7. Then, for TDT tests, taking into account three clinical subtypes and the effective number of SNPs (n = 7), the experiment-wide significance threshold required to keep type I error rate at 5% was set at *P* < 0.0024. For case-control study, considering the effective number of SNPs (n = 7), three clinical subtype, and the comparison of genotype and allele frequencies, the adjusted significance was set at *P* < 0.0012. For multi-marker association tests, haplotype analysis was only conducted when nominal association existed in the single-marker analyses and significance was corrected by 5,000 permutations using Haploview software.

The minimum statistically genetic power for TDT tests and case-control studies was calculated using the Genetic Power Calculator software (http://pngu.mgh.harvard.edu/~purcell/gpc/), taking the lowest MAF of 0.162 and assuming the prevalence of 0.05, significance level of 0.05 and odd ratio (OR) of 1.5. Under above settings, our sample showed the minimum statistically genetic power of 80.9% for TDT statistics with about 500 efficient trios, and 74.4% for 569 cases vs. 957 controls under additive model.

## Results

### Family-based association tests

#### Single-marker analyses

In the general ADHD trios, we did not find any biased transmission of any allele for any SNP (all *P* > 0.05, Table 3). Further analyses were conducted for three subtypes separately. For ADHD-I trios, TDT tests showed that the C allele of SNP rs3934492 was over-transmitted with nominal *P*-value of 0.030 (Table 3). No biased-transmission was found either in ADHD-HI or in ADHD-C trios (all *P* values > 0.05, Table 3).

**Table 3:** **TDT tests for ADHD trios and its three clinical subtypes**^**a**^

**SNP no.**	**SNP**	**All (n = 1397)**				**ADHD**-**I (n = 706)**				**ADHD**-**HI (n = 71)**				**ADHD**-**C (n = 620)**			
		**Allele**	**T:NT**	**χ**^***2***^	***P***	**Allele**	**T:NT**	**χ**^***2***^	***P***	**Allele**	**T:NT**	**χ**^***2***^	***P***	**Allele**	**T:NT**	**χ**^***2***^	***P***
SNP 1	rs4969239	A	598:577	0.38	0.540	G	297:286	0.21	0.649	A	32:28	0.27	0.606	A	280:252	1.47	0.225
SNP 2	rs4969358	A	640:637	0.01	0.933	A	332:317	0.34	0.556	C	35:32	0.13	0.714	C	285:276	0.14	0.704
SNP 3	rs6565531	G	606:602	0.01	0.908	G	335:296	2.41	0.121	A	39:33	0.50	0.480	A	267:238	1.67	0.197
SNP 4	rs8079626	G	617:598	0.30	0.586	G	337:293	3.07	0.080	A	35:34	0.01	0.904	A	270:246	1.12	0.291
SNP 5	rs3934492	C	679:615	3.17	0.075	**C**	**361:305**	**4.71**	**0.030**	C	37:31	0.53	0.467	C	281:279	0.01	0.933
SNP 6	rs9901648	G	550:542	0.06	0.809	A	285:267	0.59	0.444	G	30:24	0.67	0.414	G	253:233	0.82	0.364
SNP 7	rs4076037	G	659:630	0.65	0.419	G	351:315	1.95	0.163	A	37:26	1.92	0.166	G	282:278	0.03	0.866
SNP 8	rs8066330	T	669:652	0.22	0.640	C	347:327	0.59	0.441	T	37:28	1.25	0.264	T	305:277	1.34	0.246
SNP 9	rs4969385	C	371:357	0.27	0.604	T	193:178	0.61	0.436	C	22:17	0.64	0.423	C	171:147	1.81	0.178

*Abbreviations:TDT* transmission disequilibrium test, *ADHD* attention-deficit/hyperactivity disorder, *ADHD-I* predominant inattentive subtype, *ADHD*-*HI* hyperactive-impulsive type, *ADHD*-*C*, combined type; *T* transmitted, *NT* non-transmitted; ^a^Nominal *P* values < 0.05 are indicated in bold.

#### Multi-marker haplotype-based association tests

Haplotype analyses were only conducted for ADHD-I trios. The CA haplotype of Block 2 coded by rs3934492 and rs9901648 was over-transmitted (χ^*2*^=11.18, nominal *P*=8.0e-4, empirical *P*=0.005) and the GA haplotype was potentially under-transmitted (χ^*2*^=4.66, nominal *P* = 0.031, empirical *P*=0.207) (Table 4).

**Table 4:** TDT tests for haplotypes of Block2 with ADHD-I trios (n = 706)

**Haplotype**^**a**^	**Freq.**^**b**^	**T**:**NT**	**χ**^***2***^	**Nominal *P***	**Empirical *P***^**c**^
GA	0.440	303.9:359.5	4.66	**0.031**	0.207
CG	0.329	272.2:289.4	0.53	0.466	1.000
CA	0.229	281.3:207.4	11.18	**8.0e-4**	**0.005**

*Abbreviations:TDT* transmission disequilibrium test, *ADHD-I* predominant inattentive subtype, *T* transmitted, *NT* non-transmitted.

^a^Haplotype of block 2 coded by rs3934492 and rs9901648; ^b^Frequency of haplotypes; ^c^Empirical *P* value from Haploview estimated on the basis of 5,000 permutations. Nominal *P* values < 0.05 are indicated in bold.

### Case-control studies

#### Single-marker analyses

For allelic analyses, nominal association was displayed with ADHD for SNP rs4969385 (χ^*2*^=5.59, *P* = 0.018, OR = 1.27 [1.04-1.55]) (Table 5). What’s more, the potential confounding factors of subtypes were considered by analyses in ADHD-I and ADHD-C separately, while ADHD-HI was not analyzed due to its small sample size. Two SNPs showed association with ADHD-I: rs4969239 (χ^*2*^=6.58, *P* = 0.010, OR = 1.29 [1.06-1.58]) and rs3934492 (χ^*2*^=4.28, *P* = 0.039, OR = 1.22 [1.01-1.48]). One SNP rs4969385 showed association with ADHD-C (χ^*2*^=4.14, *P* = 0.042, OR = 1.31 [1.01-1.71]) (Table 5).

**Table 5:** **Analyses of alleles and genotypes for 569 ADHD** (**295 ADHD**-**I**, **252 ADHD**-**C**) **and controls** (**n** = **957**)^**a**^

**SNP**	**Allele**		**Alleles**		**Genotypes**										
	**1**	**2**	**2 vs. 1**		**Cases N (%)**			**Controls N (%)**				**Genotype 11 vs. 12+22**		**Genotype 11+12 vs. 22**	
			**OR (95% CI)**	***P***	**11**	**12**	**22**	**11**	**12**	**22**	***P***	**OR (95% CI)**	***P***	**OR (95% CI)**	***P***
ADHD in general															
rs4969239	A	G	1.10 (0.93-1.29)	0.261	226 (42.6)	233 (44.0)	71 (13.4)	423 (44.6)	421 (44.4)	104 (11.0)	0.366				
rs4969358	A	C	1.12 (0.97-1.31)	0.135	97 (17.3)	280 (49.8)	185 (32.9)	148 (15.6)	452 (47.6)	350 (36.8)	0.284				
rs6565531	A	G	1.05 (0.90-1.23)	0.503	82 (14.6)	252 (44.9)	227 (40.5)	146 (15.3)	441 (46.3)	366 (38.4)	0.726				
rs8079626	A	G	1.06 (0.91-1.23)	0.482	84 (15.5)	249 (45.9)	209 (38.6)	155 (16.3)	444 (46.7)	352 (37.0)	0.819				
rs3934492	C	G	1.16 (0.99-1.35)	0.055	184 (33.4)	273 (49.5)	94 (17.1)	297 (31.7)	428 (45.6)	213 (22.7)	**0.033**	1.09 (0.86-1.35)	0.490	1.43 (1.09-1.85)	**0.009**
rs9901648	A	G	1.02 (0.87-1.20)	0.811	249 (46.6)	232 (43.4)	53 (9.9)	465 (49.1)	374 (39.5)	109 (11.5)	0.283				
rs4076037	A	G	1.11 (0.96-1.29)	0.165	132 (24.5)	272 (50.5)	135 (25.0)	267 (28.1)	460 (48.5)	222 (23.4)	0.305				
rs8066330	C	T	1.08 (0.93-1.26)	0.289	146 (26.0)	279 (49.6)	137 (24.4)	220 (23.2)	496 (52.2)	234 (24.6)	0.444				
rs4969385	C	T	1.27 (1.04-1.55)	**0.018**	368 (67.6)	153 (28.1)	23 (4.2)	681 (71.4)	259 (27.1)	14 (1.5)	**0.003**	1.19 (0.95-1.50)	0.129	2.96 (1.51-5.81)	**0.001**^b^
Inattentive subtype															
rs4969239	A	G	1.29 (1.06-1.58)	**0.010**	103 (37.5)	126 (45.8)	46 (16.7)	423 (44.6)	421 (44.4)	104 (11.0)	**0.015**	1.35 (1.02-1.77)	**0.035**	1.63 (1.12-2.38)	**0.010**
rs4969358	A	C	1.21 (0.99-1.46)	0.052	55 (18.9)	146 (50.2)	90 (30.9)	148 (15.6)	452 (47.6)	350 (36.8)	0.135				
rs6565531	A	G	1.07 (0.89-1.30)	0.474	37 (12.7)	139 (47.6)	116 (39.7)	146 (15.3)	441 (46.3)	366 (38.4)	0.535				
rs8079626	A	G	1.05 (0.87-1.27)	0.612	40 (14.0)	140 (49.0)	106 (37.1)	155 (16.3)	444 (46.7)	352 (37.0)	0.611				
rs3934492	C	G	1.22 (1.01-1.48)	**0.039**	104 (36.0)	1396 (47.1)	49 (17.0)	297 (31.7)	428 (45.6)	213 (22.7)	0.091	0.82 (0.63-1.09)	0.171	0.70 (0.49-0.98)	**0.037**
rs9901648	A	G	1.00 (0.82-1.23)	0.985	130 (46.6)	124 (44.4)	25 (9.0)	465 (49.1)	374 (39.5)	109 (11.5)	0.239				
rs4076037	A	G	1.18 (0.98-1.43)	0.084	59 (21.0)	153 (54.4)	69 (24.6)	267 (28.1)	460 (48.5)	222 (23.4)	0.054				
rs8066330	C	T	1.08 (0.89-1.30)	0.412	77 (26.4)	145 (49.7)	70 (24.0)	220 (23.2)	496 (52.2)	234 (24.6)	0.526				
rs4969385	C	T	1.27 (0.99-1.62)	0.061	194 (68.3)	76 (26.8)	14 (4.9)	681 (71.4)	259 (27.1)	14 (1.5)	**0.003**	1.16 (0.87-1.54)	0.318	3.48 (1.64-7.39)	**0.001**^b^
Combined subtype															
rs4969239	A	G	1.13 (0.91-1.41)	0.281	114 (48.7)	98 (41.9)	22 (9.4)	423 (44.6)	421 (44.4)	104 (11.0)	0.497				
rs4969358	A	C	1.06 (0.87-1.30)	0.569	39 (15.7)	125 (50.2)	85 (34.1)	148 (15.6)	452 (47.6)	350 (36.8)	0.711				
rs6565531	A	G	1.04 (0.84-1.28)	0.732	41 (16.6)	104 (42.1)	102 (41.3)	146 (15.3)	441 (46.3)	366 (38.4)	0.502				
rs8079626	A	G	1.08 (0.87-1.32)	0.499	40 (17.2)	98 (42.1)	95 (40.8)	155 (16.3)	444 (46.7)	352 (37.0)	0.436				
rs3934492	C	G	1.10 (0.90-1.35)	0.341	74 (30.8)	125 (52.1)	41 (17.1)	297 (31.7)	428 (45.6)	213 (22.7)	0.103				
rs9901648	A	G	1.03 (0.82-1.28)	0.823	110 (47.2)	98 (42.1)	25 (10.7)	465 (49.1)	374 (39.5)	109 (11.5)	0.761				
rs4076037	A	G	1.06 (0.87-1.30)	0.553	66 (28.0)	108 (45.8)	62 (26.3)	267 (28.1)	460 (48.5)	222 (23.4)	0.624				
rs8066330	C	T	1.07 (0.88-1.31)	0.486	63 (25.4)	122 (49.2)	63 (25.4)	220 (23.2)	496 (52.2)	234 (24.6)	0.666				
rs4969385	C	T	1.31 (1.01-1.71)	**0.042**	158 (66.4)	71 (29.8)	9 (3.8)	681 (71.4)	259 (27.1)	14 (1.5)	0.060	1.26 (0.93-1.71)	0.131	2.64 (1.13-6.17)	**0.040**

*Abbreviations:OR* odd ratios, 95% *CI* 95% confidence interval; ^a^Nominal *P* values < 0.05 are indicated in bold; ^b^Corrected *P* values <0.0012.

Genotypic analyses also support association between above SNPs with ADHD, ADHD-I and ADHD-C (Table 5). However, only the association of rs4969385 with ADHD (*P* = 0.001, OR = 2.96 [1.51-5.81]) and ADHD-I (*P* = 0.001, OR = 3.48 [1.64-7.39]) retained significant after corrections for multiplicity, but not for ADHD-C (*P* = 0.040, OR = 2.64 [1.13-6.17]) (Table 5). Logistic regression analyses also showed retained association of *BAIAP2* with ADHD (*P* < 0.05) and ADHD-I (*P* < 0.05) after adjusting the potential effect of gender and age, but not for ADHD-C (*P* = 0.086) (Table 6).

**Table 6:** **Logistic regression analyses of genotype distribution between ADHD and controls**^**a**^

	**B**	**S.E.**	**Wald**	***P***	**OR (95% CI)**
**ADHD vs. Control (569 vs. 957)**					
Age	−0.014	0.001	149.647	< 0.001	0.99 (0.98-0.99)
Gender	−1.410	0.141	100.549	< 0.001	0.24 (0.19-0.32)
rs3934492 (11 + 12 vs. 22)	0.354	0.153	5.365	**0.021**	1.43 (1.06-1.92)
Age	−0.014	0.001	151.851	< 0.001	0.99 (0.98-0.99)
Gender	−1.429	0.142	100.615	< 0.001	0.24 (0.18-0.32)
rs4969385 (11 + 12 vs. 22)	0.850	0.377	5.092	**0.024**	2.33 (1.02-5.00)
**ADHD-I vs. Control (295 vs. 957)**					
Age	−0.011	0.001	76.853	< 0.001	0.99 (0.99-0.99)
Gender	−1.265	0.176	51.643	< 0.001	0.28 (0.20-0.40)
rs4969239 (11 vs. 12 + 22)	0.327	0.150	4.743	**0.029**	1.39 (1.03-1.86)
Age	−0.011	0.001	76.411	< 0.001	0.99 (0.99-0.99)
Gender	−1.259	0.176	51.106	< 0.001	0.28 (0.20-0.40)
rs4969239 (11 + 12 vs. 22)	0.513	0.208	6.090	**0.014**	1.67 (1.11-2.51)
Age	−0.011	0.001	76.882	< 0.001	0.99 (0.99-0.99)
Gender	−1.285	0.172	55.511	< 0.001	0.28 (0.20-0.39)
rs3934492 (11 + 12 vs. 22)	0.368	0.186	3.915	**0.048**	1.45 (1.00-2.08)
Age	−0.011	0.001	77.292	< 0.001	0.99 (0.99-0.99)
Gender	−1.297	0.173	54.534	< 0.001	0.27 (0.19-0.39)
rs4969385 (11 + 12 vs. 22)	1.023	0.412	6.174	**0.013**	2.78 (1.24-6.24)
**ADHD-C vs. Control (252 vs. 957)**					
Age	−0.015	0.002	84.927	< 0.001	0.99 (0.98-0.99)
Gender	−1.500	0.196	58.497	< 0.001	0.22 (0.15-0.33)
rs4969385 (11 + 12 vs. 22)	0.820	0.478	2.938	0.086	2.27 (0.89-5.80)

^a^Nominal *P* values < 0.05 are indicated in bold.

#### Multi-marker haplotype-based association tests

Based on the association from single-marker analyses, we performed haplotype analyses for ADHD, ADHD-I and ADHD-C. As indicated in Table 7, the CT haplotype of Block3 showed nominal association with ADHD in general (χ^*2*^=5.44, nominal *P* = 0.019, empirical *P* = 0.097). For ADHD-I, the GA haplotype of Block2 showed lower frequency (χ^*2*^=3.98, nominal *P* = 0.046, empirical *P* = 0.217) in cases than controls, while the CA haplotype showed higher frequency (χ^*2*^=5.20, nominal *P* = 0.023, empirical *P* = 0.110). For ADHD-C, the CT heplotype of Block3 showed hither frequency in cases (χ^*2*^=3.96, nominal *P* = 0.047, empirical *P* = 0.215). However, none of above association remained significant after permutations.

**Table 7:** Haplotype analyses for 569 ADHD versus 957 controls

**Haplotype**		**ADHD (n = 569)**			**ADHD**-**I (n = 295)**			**ADHD**-**C (n = 252)**		
		**χ**^**2**^	**Nominal *P***	**Empirical *P***^**c**^	**χ**^**2**^	**Nominal *P***	**Empirical *P***^**c**^	**χ**^**2**^	**Nominal *P***	**Empirical *P***^**c**^
**Block2 **^**a**^	**GA**↓	3.31	0.069	0.307	3.98	**0.046**	0.217	0.76	0.384	0.890
	**CG**	0.15	0.696	0.993	0.01	0.909	1.000	0.11	0.736	0.996
	**CA**↑	3.26	0.071	0.312	5.20	**0.023**	0.110	0.56	0.456	0.936
**Block3 **^**b**^	**TC**	1.11	0.291	0.781	0.66	0.419	0.902	0.49	0.484	0.948
	**CC**	0.49	0.482	0.937	0.33	0.561	0.967	0.63	0.427	0.916
	**CT**↑	5.44	**0.019**	0.097	3.47	0.062	0.274	3.96	**0.047**	0.215

^a^Haplotype of block 2 coded by rs3934492 and rs9901648; ^b^Haplotype of block 3 coded by rs8066330 and rs4969385; ^c^Empirical *P* value estimated on basis of 5,000 permutations; ↓: the frequency in ADHD cases was lower than that in controls; ↑: the frequency in ADHD cases was higher than that in controls. Nominal *P* values < 0.05 are indicated in bold.

## Discussion

Our results showed that *BAIAP2* was associated with childhood ADHD of Chinese Han descent, especially for the predominantly inattentive type (ADHD-I). Analyses for case-control studies indicated different alleles and genotypes distribution between ADHD and controls, and the association was only retained for ADHD and ADHD-I after multiple corrections or adjusting the confounding effect of gender and age, but not for ADHD-C. From TDT tests for trios, the significant association was only indicated for ADHD-I from haplotype analyses.

In some extent, our current findings are consistant with previous reports. Ribasés et al. [16] have analyzed six functional candidate genes showing differential expression between hemispheres in ADHD and normal controls, but only found an association between *BAIAP2* with ADHD. However, in their study, the association of *BAIAP2* with ADHD was only observed in adults, which has also been replicated in an independent population, but not children. The possible explanation for this discrepancy may be that the genetic load for ADHD children may be lower than ADHD adults, while the sample size in the study by Ribasés et al. [16] may be not enough to detect the relatively weak association in ADHD children. Further investigation in ADHD adults of Chinese Han descent especially by follow-up studies may promote our understanding of the explicit effect of *BAIAP2* on ADHD.

In our present study, the SNP rs4969385 was the only associated one with ADHD and ADHD-I after corrections for multiple testing. This SNP has also been reported in adult samples by Ribasés et al. [16]. This replication suggested further exploration of this SNP and its related functional variants in the predisposition to ADHD. However, we note that the direction of the effect in our current study was opposite to the one observed by Ribasés et al. [16]. In our study, the minor ‘T’ allele showed risk for ADHD, while the major ‘C’ allele did in Ribasés’s report. Previous studies has also reported similar phenomenon between different ethnicities [29,30]. In addition, another SNP rs8079626, showing none association in our current study, has been reported to be of nominal association with adult ADHD in German sample by Ribasés et al. [16]. In their study, the ‘A’ allele showed higher frequencies in German adult ADHD sample than controls. In our study, for the ‘G’ allele, the transmission was more than non-transmission from TDT tests and its frequency was higher in ADHD probands versus controls, although not achieving statistically significant difference. When checking the LD between rs8079626 with the best two SNPs of our current analyses, we found its low LDs with rs3934492 (*D*’ = 0.231, *r*^*2*^ = 0.04) and rs4969385 (*D*’ = 0.072, *r*^*2*^ = 0.002). So we can conclude that the SNP rs8079626 was not associated with ADHD in Chinese Han subjects. This discrepancy may be greatly due to the ethnic difference, that its allele distribution in our current analyses of Chinese Han subjects (62.8% of G, 37.2% of A) was adverse to that of German samples (31.0% of G, 69.0% of A) from the study by Ribasés et al. [16]. This adverse distribution is according with the Hapmap database (62.2% of G for CHB, 28.3% of G for CEU). Taken together, it is very important to consider potential pathogenic genetic variants in different ethnic populations for ADHD etiological studies.

Another interesting finding is the specific association of *BAIAP2* with ADHD-I subtype. Three subtypes of ADHD were defined in DSM-IV including predominantly inattentive, predominantly hyperactive/impulsive and the combined subtypes [31]. A growing body of literature has addressed that inattentive and combined subtypes may be distinct disorders [32,33]. Attention-deficit disorder (ADD; inattentive, without hyperactivity) was different from ADHD on genetic basis, comorbidity, related brain region, etc. (for a review, see [33]). These findings have some effect on the revision of ADHD subtypes in DSM-V [34]. Our previous studies on molecular genetics have revealed the potential subtype-specific genes associated with ADHD in the Chinese Han population. *COMT*, *5*-*HT1B*, *MAOA*, *CHRNA4*, *SYP* and *DDC* exhibited associations primarily with ADHD-I, whereas *HTR2C*, *5*-*HT1D*, *ADRA2C*, *DRD3* and *NET1* were associated mainly with ADHD-C [24,35-38]. The family-based study of *NET1* and ADHD strongly suggested that the haplotype blocks within different regions of *NET1* show divergent association based on sex and subtype [39]. Taken the current finding together with previous reports, considering subtypes in ADHD genetic studies is very important to reduce the heterogeneity and may help us to explore the hidden existed genes associated with ADHD.

Our findings need to be considered in light of some limitations. Firstly, twenty-seven genes have been identified involving in the left-right asymmetric cortical development in humans [15]. We only included *BAIAP2* in this study and whether other genes played the role for the pathogenesis of ADHD remained unclear. Secondly, we have set MAF > 0.25 for tag SNPs selection and included an additional reported SNP rs4969385 with MAF of 0.162. We could not preclude the rare genetic contribution of uncommon SNPs (MAF <0.162) to the occurrence of ADHD. Thirdly, we have not screened those parents for ADHD diagnosis that we could not explain the genetic mechanisms of *BAIAP2* in the etiology of ADHD clearly. Fourthly, ADHD was associated with high rates of comorbidity including dyslexia (reading disorder). In the existing literature, evidence has support the common risk neurobiological phenotype of atypical cerebral asymmetry (ACA) and shared ACA genes for ADHD and dyslexia [40]. The diagnosis instrument as described above in the present study lacked the ability to differ ADHD from dyslexia. Lastly, we did not collect data on the handedness, which is also strongly correlated with cerebral asymmetry. Then, we could not preclude the confounding effect of the diagnosis of dyslexia and handedness on our observed associations.

## Conclusions

The present family-based and case-control association studies in our Chinese Han populations provide further evidence for the role of *BAIAP2* in the predisposition to ADHD, especially ADHD-I. Further studies should investigate the involvement of hemispheric asymmetry genes modulating the difference of left-right hemisphere in ADHD and related cognition.

## Abbreviations

ADHD: Attention-deficit/hyperactivity disorder; ADHD-I: ADHD predominantly inattentive type; ADHD-HI: ADHD predominantly hyperactive/impulsive type; ADHD-C: ADHD combined type; SNP: Single nucleotide polymorphisms; CDIS: Clinical Diagnostic Interview Scale.

## Competing interests

The author’s declare that they have no competing interests.

## Authors’ contributions

LZH and QQJ designed the study. LL, SL, LZH and LHM participated in data collection and experiments. LL, LZH and WLP participated in the statistical analyses and interpreted the results. LL, SL and QQJ drafted the manuscript. WYF supervised the whole study and revised the manuscript. All authors read and approved the final manuscript.
